# Developing Measures of Immersion and Motivation for Learning Technologies in Healthcare Simulation: A Pilot Study

**DOI:** 10.30476/JAMP.2022.95226.1632

**Published:** 2022-07-01

**Authors:** CHRIS JACOBS, JACOB M. RIGBY

**Affiliations:** 1 Swindon Academy, Great Western Hospital, Swindon, UK; 2 University of Bath, Swindon, UK; 3 School of Geographical Sciences, University of Bristol, University Road, Bristol, BS8 1SS, UK

**Keywords:** Patient simulation, Immersion, Virtual reality, Educational assessment

## Abstract

**Introduction:**

Medical education has benefitted from the introduction of new technology within recent years. Immersive devices, such as, 360-degree films and virtual reality have become new ways of simulating clinical experiences. The aim of the study was to validate and test reliability of a new measure of engagement.

**Methods:**

A between-participants design of 2 groups viewing a clinical consultation on a 360-degree headset or 2D monitor was conducted following computer random allocation of 40 healthcare professionals recruited from scheduled teaching. Twenty-three were assigned to 360-degree and 17 to 2D Medias. Adapted Immersion Experience Questionnaire (AIEQ) and Abridged Intrinsic Motivation Inventory (AIMI) were modified to match factors relating to clinical encounters. AIEQ and AIMI were utilised as the data collection tool by each group following video viewing. Spearman’s rank correlation was used to assess relationship between immersion and motivation. Comparisons between 360-degree and 2D media responses were made using Wilcoxon’s signed ranks test. Internal reliability coefficients of adapted measures were calculated with Cronbach alpha scores.

**Results:**

Total immersion scores were statistically higher in those experiencing 360 (p<0.05), with a median difference of 14.50 (95% CI 6.50-22.00). A positive correlation existed between the total AIEQ and total score of the AIMI in both groups (r_s_ =0.88, n=17, p<0.001). Internal consistency and reliability was demonstrated with a high Cronbach alpha score for the AIEQ (α= 0.91). AIMI subscale alpha value was also high at (α= 0.95) which shows the measures to be of high internal reliability.

**Conclusions:**

Adaptation and validation of existing measures for use in healthcare education can be used to quantify levels of immersion and motivation. Standardising measures for use in evaluating new Technology Enhanced Learning is a step to aid understanding on how we develop these tools in medical education and how we might learn from immersive technology.

## Introduction

Learning in the clinical environment is often described as experiential, whereby Kolb’s learning theory considers the transformation of an experience to a construct that can be further tested and refined ( 1 ). Simulation-based education are activities that replicate real events and give opportunities for learners to participate in either an active or passive role. There are simulated experiences in healthcare education that can provide levels of fidelity (from low to high) and modalities that can include patient manikin, virtual reality, actors, and simulation trainers ( 2 ). For example, healthcare educators might aim to mimic a clinical encounter in simulation to provide a learning experience with the use of immersive 360-degree video (360). Innovative Technology Enhanced Learning (TEL) provides an opportunity to learn from a digital resource ( 3 , 4 ), which can be both remote from origin and experienced anytime.

As new TEL expands rapidly, it allows learners to consume digital material in various formats asynchronously to others and via different electronic devices. Electronic learning is the delivery of knowledge and skills via digital resources ( 5 ). The E-Learner typically navigates these mediums in a capacity for autonomous learning that is part of a goal orientated self-actualised behaviour ( 4 ). Health care professionals (HCP) can access this diverse content from mobile devices or computers. In a study conducted in a Canadian medical school reported over 85% of medical students regularly using mobile phones for education purposes ( 6 ).

A United Kingdom National Health Service review entitled ‘preparing the healthcare workforce to deliver the digital future 2019 review’ was predicated on pre-suppositions. In particular, 

*‘There is remarkable potential for digital healthcare technologies to improve accuracy of diagnoses and treatments, the efficiency of care, and workflow for healthcare professionals, but implementation must only be carried out when there has been robust clinical validation.’* ( 7 ).

Immersion can be defined as a psychological state of engagement with a task or medium that varies in depth ( 8 ), and where the presence or connection to the physical world is inversely associated with task engagement ( 9 ). Technology allows viewers to experience a recreation of the clinical environment whilst remaining remote or asynchronous to the event. For example, a 360 video of a clinical consultation or a virtual reality (VR) simulated patient ( 10 ). The level of interactivity with the learning environment can exist on a continuum from less to more interactive, with 360 being a passive consumption of media ( 11 ). The designer of the 360 resources can, however, edit and adapt to learning needs of a group and provide financial benefit through scalability ( 12 ). A significant number of authors conclude that immersion was an important feature of education using 360 or VR ( 13 - 17 ).

Learning, in part, is determined by a person’s motivation states and how their self-image projects a readiness to learn. Motivation to learn holds intrinsic value with a personal self-directed pay off. This being self-governed by a need know: why, what and how? ( 18 ) These assumptions to principles of learning forms part of the andragogy of learning. Learning theory that arguably derives a curriculum from HCP self-actualisation and competence is based on constructs and assumptions, creating a challenge to measure and understand the homogenous and heterogenous concepts within these domains of learning. Motivational states relate to numerous learning approaches researched ( 4 , 19 - 21 ) and have been described as intrinsic and extrinsic in nature.

Healthcare educators would benefit from understanding how TEL might influence a user’s immersion in or engagement with learning media and thus consider how this affects learning. Given a learner’s motivational state could influence the user’s engagement with technology and content, there is implication that quantifying this alongside immersion will enable greater understanding. Having a validated measure that reliably quantifies user experience whilst consuming medical based media would allow both learner and educator to reflect on how we might learn. Currently, there is a lack of standardised measures that allow assessment of engagement and comparison to different media in medical education ( 22 ).

Although we can assume that immersion is similar in using TEL in medical education to that of gaming, there are underlying motivational and outcome differences. This highlights the importance of testing metrics for their validity for use in the clinical environment.

This study aims to assess engagement and motivation in this context, and describes the adaptation and validation of a questionnaire for immersive technology in medical education. Additionally, authors will aim to demonstrate reliability to ensure reproducibility. 

### Review of existing measures for engagement in video games and motivation

User immersion and related concepts have been a focus for video games researchers for several decades, with numerous measures being developed ( 23 ). Researchers in this area often regard immersion as a state that incorporates enjoyment and real-world dissociation to heighten gaming experience. Jennett, et al. ( 9 ) conceptualised that immersion encompasses dimensions of presence, cognitive absorption and flow. These factors are applicable to non-participatory video as though, a linear media, users can experience the 3 factors ( 24 ). Other psychometrics exist that measure, for example, Core Elements of the Gaming Experience. However, the domains of interest are too game-centric as feature scales on puppetry, control, and gameplay ( 25 ). Thus, less suitable to this study. 

The IEQ benefits from quantifying emotional connection, dissociation from the real-world environment, and resumed task engagement. The objective measure was constructed by comparing a control group and a gaming group and measuring time to re-engagement following the end of the task. Additionally, eye movement tracking was used to ensure participant remained focus on the gaming task as a method of reliability check. Furthermore, measure results were matched to show high correlation with total immersion score of IEQ and time to re-engage. Most IEQ questions are not specific to media content and are transferable as it is a measure of an experience. 

A questionnaire that evaluates the internal motivations orientated to a task was developed by Ryan ( 26 ) and the full instrument is a 22-item measure that includes the domains of: perceived choice, pressure, enjoyment or interest, effort, and value. The experimental work established that the enjoyment subscale had greatest relevance to intrinsic motivation and hence, has the greater proportion of questions. The value subscale has been used in internalisation studies and reflects aspects of self-regulation. Both the full measure and abbreviated versions have shown validity in numerous studies ( 27 - 29 ). The Intrinsic Motivation Inventory (IMI) was abridged (AIMI) to a 12-item measure that included subscales on enjoyment and value in this study with the assessment of reliability.

## Methods

### Adapted immersive experience questionnaire development

The multi-dimensional measure of engagement is based on existing video game research of Jennett, et al. and their development of the Immersive Experience Questionnaire (IEQ) ( 9 ) and adapted to a simulated HCP interaction: the adapted immersive experience questionnaire (AIEQ). Similar work on validating IEQ to film incorporates the principles explored with relationship to media on differing screen size ( 30 ). As adaptations to the questionnaire are required for use in the clinical setting a process of content validity is needed. Prior exploratory factor analysis on IEQ ( 9 , 30 ) informs of existing construct validity.

The research group in 360-degree video at Great Western Hospital (GWH) undertook review of 31 questions of original IEQ to correct game-specific words and alter tense for correct grammar relating to watching a clinical consultation. Eight questions were not applicable to the study, for example, ‘how much did you want to win the game?’. Additionally, if rewording was not possible, then a replacement question would keep the theme applicable to immersive media. Following this work, the AIEQ consisted of 23 items using a 1-5 Likert scale and 1 item using a 1-10 Likert scale (see table 1). The AIEQ was piloted with a small sample of 4 medical students.

**Table 1:** Development of AIEQ (Adapted Immersion Experience Questionnaire) from original experimental IEQ (Immersion Experience Questionnaire). Commentary is provided to describe process of adaptations

Original Question	Adapted Question (changes in bold)	Commentary
I felt that I really empathised/felt for with the game.	I felt that I really empathised/felt for with the **patient**.	*Edited to place focus on clinical encounter and central importance of patient.*
I did not feel any emotional attachment to the game.	I did not feel any emotional attachment to the **patient**.
I was interested in seeing how the game’s events would progress.	I was interested in seeing how the **consultation** would progress.
It did not interest me to know what would happen next in the game.	It did not interest me to know what would happen next during the consultation.
I was in suspense about whether I would win or lose the game.	Omitted	*Omitted as there is not a game to succeed or lose at.*
I was not concerned about whether I would win or lose the game.	Omitted
I sometimes found myself to become so involved with the game that I wanted to speak to the game directly.	I sometimes found myself to become so involved with the consultation that I wanted to speak **to the individuals**.	*Re-iterated focal point of consultation.*
I did not find myself to become so caught up with the game that I wanted to speak to directly to the game.	I did not find myself to become so caught up with the **consultation** that I wanted to speak to the **individuals**.
I enjoyed the graphics and imagery of the game.	I enjoyed the **visuals** of the **consultation**.	*360 video is a visual experience, and this question quantified the participants experience and removed imagery as this may have alternative meanings.*
I did not like the graphics and imagery of the game.	I did not like the **visuals** of the **consultation**.
I enjoyed playing the game.	Omitted
Playing the game was not fun.	Omitted
The controls were not easy to pick up.	The controls were not easy to pick up.	*Enjoyment of a game was removed questions as motivation were included in a further study. Also, the reversed question wording differed. *
There were not any particularly frustrating aspects of the controls to get the hang of.	There were not any particularly frustrating aspects of the controls to get the hang of.
I became unaware that I was even using any controls.	Omitted
The controls were not invisible to me.	Omitted	*Removed questions that had further details on controls as 360 video is linear and passive. This is quite different to a computer game.*
I felt myself to be directly travelling through the game according to my own volition.	Omitted	
I did not feel as if I was moving through the game according to my own will.	Omitted	
It was as if I could interact with the world of the game as if I was in the real world.	It was as if I could interact with the **environment** as if I was in the real world.	
**Interacting with the world of the game did not feel as real to me as it would be in the real world**.	Interacting with the environment did not feel as real to me as it would be in the real world.	*Game world reworded as environment *
I was unaware of what was happening around me.	I was unaware of what was happening around me.	
I was aware of surroundings.	I was aware of surroundings.	
I felt detached from the outside world.	I felt detached from the outside world.	
I still felt attached to the real world.	I still felt attached to the real world.	
At the time the game was my only concern.	At the time the consultation was my only concern.	*Game substituted for consultation*
Everyday thoughts and concerns were still very much on my mind.	Everyday thoughts and concerns were still very much on my mind.
I did not feel the urge at any point to stop playing and see what was going on around me.	I did not feel the urge at any point to stop **watching** and see what was going on around me.	*Verb for task change*
I was interested to know what might be happening around me.	I was interested to know what might be happening around me.
I did not feel like I was in the real world but the game world.	**I wanted to learn more on the patient outcome following the consultation**.
I still felt as if I was in the real world whilst playing.	**I was not interested in learning more on the patient’s outcome following the consultation**.	*Game narratives are continued when playing is restarted. Unlike clinical encounters whereby future state of patient’s are considered by clinicians.*
To me it felt like only a very short amount of time had passed.	To me it felt like only a very short amount of time had passed.	
When playing the game time appeared to go by very slowly.	When **watching** the **consultation** time appeared to go by very slowly.	
How immersed did you feel? (10=very immersed; 0=not at all immersed)	How immersed did you feel? (10=very immersed; 0=not at all immersed)	*Finally, reworded to correctly reflect task.*

Comparison of experience following consumption of the same media on two different technologies was the validation experiment analysing the effect of immersion and tool development.

### Reliability and validity analysis

After the development of the AIEQ the scores were analysed to test the internal validity of the questionnaire using Spearman’s rank correlation. Also, reliability analysis with comparing Cronbach alpha was undertaken to ensure questionnaire reconstruction maintained a high reliability that Jennett, et al. described ( 9 ).

### Study design

This study used a between-participants design, with two conditions: 1. Viewing a 2D medical consultation on a laptop screen, and 2. Viewing the same consultation in 360 using a Samsung Galaxy Gear VR, where participants had the freedom to look around. The dependent variables were immersion score (assessed by the AIEQ) and intrinsic motivation score (assessed by the AIMI). 

### Participants

The measures were completed by 40 participants. This was a pool of different HCP, including: medical students, junior doctors, and physician associate students, see table 2. These were recruited from GWH, Swindon, and University of West of England. Sampling of participants took place during core clinical teaching sessions. Thirty were female (75%), and 10 were male (25%). Twenty-eight participants (72%) were in the 20-25 age range, and the remaining 11 participants (28%) were aged 25-30. 

**Table 2:** Frequency and relative proportion according to HCP (Health care professionals) status

HCP background	Frequency	Relative proportion
Doctor	5	12.5%
Medical student	17	42.5%
Physician associate	2	5%
Physician associate student	16	40%

### Inclusion criteria:

• Age ≥18
• Student status as a healthcare professional
• Qualified healthcare professional and undertaking further education related to clinical training.

### Exclusion criteria:

• Pilot participants who viewed the video.
• Qualified status in healthcare setting and no longer in a training programme.

Computer generated randomisation occurred to allocate to 2 groups: 360 arm and conventional 2D arm. This was a simple randomisation following consent with the participants initially blinded to video format.

**Figure 1:**
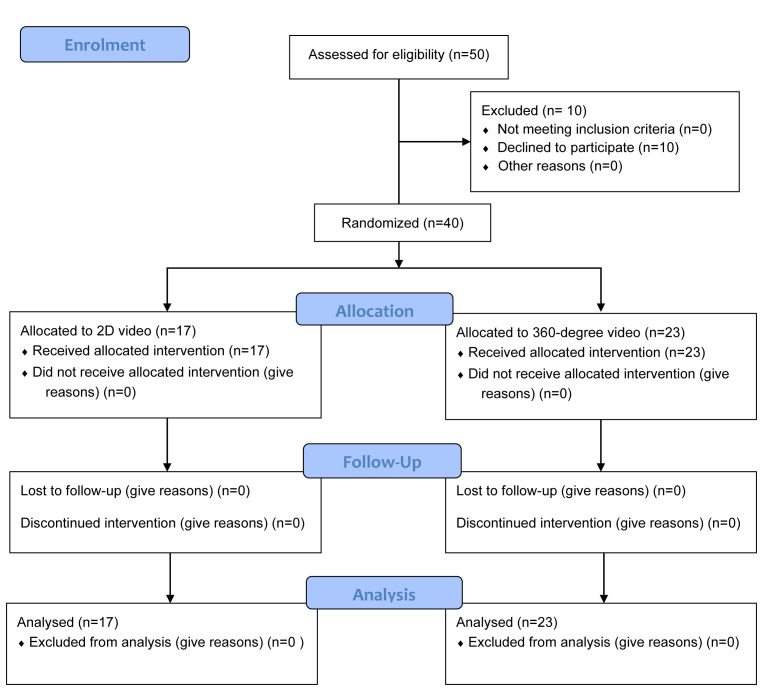
CONSORT flow diagram of trial

Flow diagram of parallel randomised trial CONSORT diagram outlines the process, in Figure 1.

There was no reward for inclusion in the study.

### Ethical Consideration

University of Bath psychology departmental ethics committee approved the project in 2019 (ref 18-092). Participant information was anonymised and stored securely in accordance with the General Data Protection Regulations United Kingdom (GDPR).

### Materials

Stimuli for the study consisted of two versions of a consultation between a General Practitioner (GP) and a trained clinical actor presenting with abdominal pain in a GP surgery. The first was a 360-degree video of this session, recorded on a 360-camera positioned on a tripod centrally in the room. This would give the viewer a virtual position placed in the middle of the room. The GP moved around the room during the consultation. The second was a 2D version of the same session, created from the original 360-degree recording: the video was edited to insert a title, and to create a 2D version of the consultation mapping the movements of the actors to mimic camera panning to give content similarity between formats. Both videos were approximately seven minutes in duration. 

Data were collected from the participants using an online form, which consisted of participant information followed by digital consent form and AIEQ and AIMI. A Samsung Gear 360 VR headset was provided with a laptop to complete measures following watching the consultation and to display the flat screen (2D) film version.

### Procedure

The experiment was conducted in a partitioned lab that divided a simulation suite to provide a quiet area with no visual or sound distractions. A researcher facilitated the experiment, which began by explaining the procedure to the participants and helping them familiarise themselves with the Samsung Gear VR controls. The headset controls allowed users to pause the video using a button on the side. This functionality was available to those viewing the consultation in 2D using the laptop. The researcher also explained that nausea wearing a VR headset can occur and demonstrated how to remove the device. Before commencing, the participants completed a consent form. The participants assigned the 360-video condition were seated on a swivel chair away from any obstacles, and participants assigned the 2D condition were seated in front of the laptop. The participants then viewed their assigned video condition and completed the AIEQ and AIMI immediately afterwards with data recorded on Google survey tool with a provided laptop. Finally, they completed a questionnaire to collect demographics of education background and whether they were an HCP student or qualified and in training. One member of the research team remained present behind the partition to be available for any difficulties experienced. 

### Statistical analysis plan

A test for kurtosis and histogram plotting determined normality of data. From previous studies non-parametric dataset is predicted using IEQ. Likert scales will be treated as continuous data with Wilcoxon’s signed rank for non-parametric data, Spearman’s rank for correlation, and Cronbach’s Alpha for reliability will be used for statistical analysis of scores. These will be undertaken using StatsDirect statistical software (version 3.3.5).

StatsDirect (version 3.3.5) sample size calculation is based on prior work on IEQ with estimated difference in means of groups of 10.00 and estimated standard deviation of 10.00. ( 30 ) Additionally, a probability of 80% of correctly detecting a real effect (power) and 5% probability of incorrectly rejecting hypothesis (alpha) were assumed. The unpaired two sample estimates a minimum sample of 17 in each group. 

## Results

### Descriptive findings

No missing data was found, and all 40 participants’ responses were analysed. Through process of computerised random group allocation, 17 were randomised to the 2D arm and 23 to 360 arm. 

The 40 responses for the AIEQ were scored with score inversion of the counter negative questions. A test for kurtosis and histogram plotting implied non-normally distributed data. The final sum was calculated with a potential maximum of 120.00, total immersion score (TI Score). 2D arm median TI score was 65.00 (IQR=59.00-70.00) and 360 median TI score 80.00 (IQR=75.00-85.00). Maximum TI score for 2D was 83.00, compared with 92.00 for 360 group. See table 3 for descriptive findings.

**Table 3:** Results from 2 groups. Key AIEQ- Adapted Immersion Experience Questionnaire, AIMI- Abridged Intrinsic Motivation Inventory, 2D- flat screen monitor, 360- 360-degree headset

Variables	AIEQ score 2D	AIEQ score 360	AIMI score 2D	AIMI score 360
Valid data	17	23	17	23
Missing data	0	0	0	0
Standard deviation	8.86	8.07	13.20	10.71
Standard error of mean	2.14	1.68	3.20	2.233
Lower 95% CL of mean	61.62	75.85	49.44	63.54
Maximum	83.00	92.00	78.00	84.00
Upper quartile	70.00	85.00	61.00	76.00
Median	65.00	80.00	58.00	69.00
Lower quartile	59.00	75.00	50.00	61.00
Interquartile range	11.00	10.00	11.00	15.00
Minimum	52.00	65.00	23.00	43.00
Range	31.00	27.00	55.00	41.00
Centile 95	83.00	92.00	78.00	82.00
Centile 5	52.00	65.00	23.00	52.00

### Outcomes

**Figure 2:**
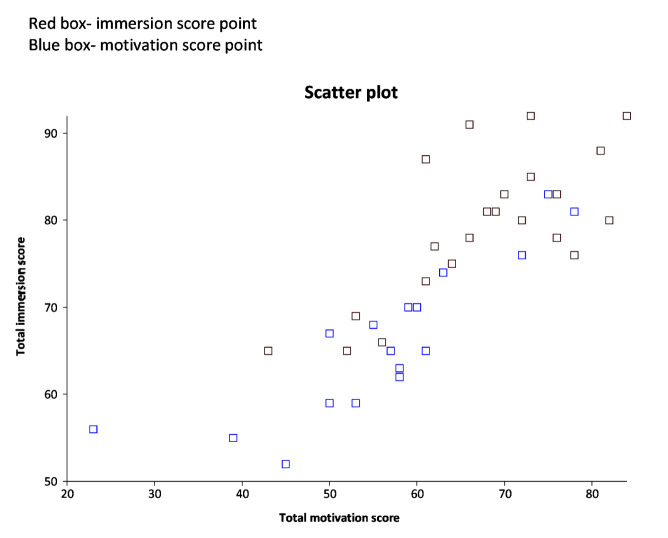
Scatter plot representing variable of total immersion score and total motivation score

A Wilcoxon signed-rank test indicated that the immersion scores were statistically higher in those experiencing 360 (p<0.05), with a median difference of 14.50 (95% CI 6.50-22.00). A positive Spearman Rank correlation existed between the TI score and total score of the AIMI in both groups (r_s_ =0.88, n=17, p<0.001) as represented in Figure 2.

Additionally, a positive Spearman Rank correlation existed between Q.21 that asked users to rate their immersion, Likert score 1-10, with TI score (r_s_ =0.87, n=40, p<0.001). This positive relationship establishes a degree of criterion-related validity on AIEQ for an index of immersion.

### Measure Reliability

To screen for internal consistency reliability within 23-item AIEQ an overall alpha was calculated with a value of 0.91, which represents high consistency as above accepted 0.8. Individual questions were analysed for possible deletion; however, the maximum change for any question removal was -0.008, which would not significantly improve the alpha score. Participant numbers were too small to conduct an exploratory factor analysis to investigate if a predictive subset of variables existed. 

AIMI alpha value was also high (α= 0.95), which shows the 12-item measure to be of good reliability. Again, the highest change whether any question to be removed was -0.008 and this would have no statistical impact on the alpha score found. Again, participant numbers were too low to analyse any meaningful subsets of variables.

## Discussion

Innovative technology that transports the user into a clinical setting creates an increase engagement with the subject matter. AIEQ demonstrated a significant score difference when audiences viewed the same clinical consultation on a 360 headset. Factors influencing this included the real-world dissociation and captivation that 360 offers over conventional 2D audio-visual experiences. Rigby, et al. found similar findings when participants watched films on differing screen sizes with the larger screens demonstrating greater participant immersion ( 30 ).

In a study that utilised 360 headsets to measure the effects of motivation and competency found higher motivation and self-reported learning competency (p<0.05) ( 31 ). The tools to measure these findings reported no testing of the validity or reliability of the instruments. Similarly, in studies utilising 360 for simulation of palliative care, paediatric scenarios, and situational awareness indicated high degrees of immersion and positive student experience ( 32 - 34 ). However, in each of these studies the measures to capture the experience lacked a process of assessing the suitability and reliability of the questions asked. 

Higher levels of motivation correlated with higher immersion scores in this study and this could represent a paradox, in that, does increasing immersion motivate a learner or vice-versa. Further studies to evaluate participant experiences in the motivational and engagement states of individuals in learning could be valuable to explore this. This study considered values and enjoyment attributable to intrinsic motivation drivers and as we aim to encourage group learning and working, scope exists to consider evaluating extrinsic motivational factors and communities of practice theory ( 4 ).

Although statistically significant findings were demonstrated, a limitation to this study was the low participant numbers and caution applied to how results can be interpreted. This study represents exploratory work that begins the foundations of creating methods for capturing user experience with visualisation technology. Unequal group sizes were a product of randomisation, which may have influenced the results. However, unequal group size in randomised trials can be expected and moderate disparities are accepted ( 35 ).

Interpreting AIEQ with differing TEL should note that users of new technology can experience a feeling and emotion of awe ( 36 ). This can influence users’ responses with a positivity towards using new technology, which may be temporary and mask any opposing thoughts. 

Future work could explore this by including qualitative information that would supplement the data collection on any metrics ( 6 ).

Given the lack of standardisation in measures to quantify user experience with TEL or participants in simulation ( 37 ) this study evidences that existing validated measures can be adapted for clinical use. Learning and video gaming can be considered as not mutually exclusive as the gaming research community have shown learning occurs whilst gaming ( 38 ). Future work could expand on this by performing more in-depth validation exercise of the AIEQ, such as a factor analysis as in the original IEQ and the version adapted for video media. While the current study demonstrates this measure as appropriate for clinical use, this could lead to a greater understanding on the underlying structure of the measure providing additional reassurances of its validity.

## Conclusion

TEL will continue to develop and advance into medical education and how we integrate this in HCP curricula matters. Possessing a greater understanding on how these new technologies affect the user experience can bring us a step closer to interpretation within the context of the complexity of learning.

This study evidences the reliability and validity on adapting instruments to measure engagement and immersion.

Medical education could benefit from borrowing and adapting more of the existing measures employed in assessing participant engagement and other learner domains as this can have numerous applications for learners, educators and developers.

## Authors' contribution

Ch.J, J.M.R contributed to the conception and design of the work; the acquisition, analysis, or interpretation of data for the work. All Authors contributed in drafting and revising the manuscript critically for important intellectual content. All authors have read and approved the final manuscript and agree to be accountable for all aspects of the work in ensuring that questions related to the accuracy or integrity of any part of the work are appropriately investigated and resolved.

## Conflict of Interest:

None declared.
